# Comparison of Pediatric and Adult Tonsillectomies Performed by Thermal Welding System

**DOI:** 10.1155/2013/265105

**Published:** 2013-11-28

**Authors:** Tolga Ersözlü, Yavuz Selim Yıldırım, Selman Sarica

**Affiliations:** ^1^Department of Otorhinolaryngology Head and Neck Surgery, Elbistan State Hospital, 46300 Kahramanmaras, Turkey; ^2^Department of Otorhinolaryngology and Head and Neck Surgery, Faculty of Medicine, Bezmialem Vakif University, Adnan Menderes Bulvarı, Vatan Caddesi Fatih, 34093 Istanbul, Turkey; ^3^Department of Otorhinolaryngology Head and Neck Surgery, Afşin State Hospital, 46300 Kahramanmaras, Turkey

## Abstract

*Objective.* To compare pediatric and adult age groups in terms of postoperative bleeding and pain following tonsillectomy performed by thermal welding system (TWS). *Method.* The study consisted of 213 patients, of whom 178 were children and 35 were adults. The mean age of the pediatric patients (81 girls and 97 females) was 6.7 ± 2.4 years (range 3–13 years) and the mean age of the adults (20 males and 15 females) was 21.8 ± 7.07 years (range 15–41 years). All of the patients were evaluated in terms of postoperative bleeding and pain following tonsillectomy performed by TWS. *Results.* Bleeding was detected in the late postoperative period in 11 pediatric and 7 adult patients and of them 2 pediatric and 3 adult patients controlled under general. Postoperative bleeding was significantly less prevalent in the pediatric age group compared to the adult age group (*P* = 0.04). Likewise, postoperative pain was significantly less prevalent in the pediatric age group as compared to the adult age group (*P* < 0.001). *Conclusion.* Both postoperative bleeding and pain following tonsillectomy performed by TWS were more prevalent in the adult age group compared to the pediatric age group.

## 1. Introduction

Tonsillectomy is the most common surgical procedure performed in the ear, nose, and throat practice. It is most frequently performed via cold dissection both in the pediatric and adult age group worldwide. Many hot dissection methods have been defined as an alternative to the cold dissection. Hot dissection methods include bipolar and/or monopolar electrocautery, radiofrequency, harmonic scalpel, coblator, and thermal welding system (TWS) [1]. Previous studies have reported and compared the outcomes of radiofrequency, TWS, cold knife, or monopolar electrocautery in terms of posttonsillectomy bleeding and pain [2]. Thermal welding is the technique that simultaneously uses heat and pressure to provide coagulation. In a study conducted on adults, tonsillectomies performed by TWS and bipolar electrocautery were compared in terms of intraoperative bleeding, operation duration, postoperative pain, time to regain normal diet, and postoperative bleeding [3, 4]. Multiparametric studies comparing a few methods have been conducted also in the pediatric age group [5]. However, studies comparing adult and pediatric patients in terms of postoperative bleeding and pain following tonsillectomy performed by TWS are limited. The aim of the present study was to investigate and compare the postoperative bleeding and pain following tonsillectomy performed by TWS in the pediatric and adult patients.

## 2. Materials and Method

The present prospective study included 213 pediatric and adult patients that underwent only tonsillectomy and/or adenotonsillectomy under general anesthesia by thermal welding device in Elbistan State Hospital Ear-Nose-Throat clinic between February 2008 and June 2012. Of the patients, 178 were children aged between 3 and 13 years and 35 were adults aged between 15 and 41 years. The indications for tonsillectomy were recurrent tonsillitis and/or tonsillar hypertrophy in the pediatric patients and chronic tonsillitis in the adult patients.

Patients with history for penicillin allergy, coagulation disorder and/or abnormal elevated prothrombin time, and activated partial thromboplastin time were excluded from the study. All of the patients were operated by the same surgeon (Togla Ersözlü) under general anesthesia with endotracheal intubation. One hour prior to the surgical procedure, the pediatric patients were given 500 mg ampicillin/sulbactam intravenously, whereas the adult patients were given 1000 mg ampicillin/sulbactam intravenously. Both pediatric and adult patients received 0.5 mg/kg methylprednisolone intraoperatively. After the surgery, patients in the pediatric age group received amoxicillin/clavulanate and paracetamol suspension two times a day for ten days and the patients in the adult age group received amoxicillin/clavulanate and paracetamol tablets two times a day with ten days.

### 2.1. Surgical Technique

**Figure 1:**
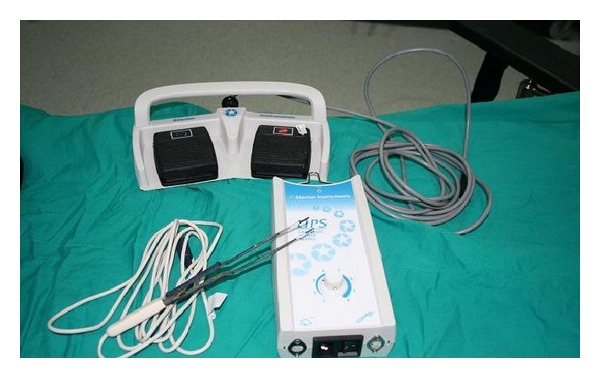
Thermal welding system (TWS) device.

TWS consists of a single-use probe (ENTceps), double-controlled foot switch, and a universal power supply (UPS). The energy from the universal power supply turns into thermal energy in the heating wire (nichrome) at the distal end of the thermal welding probe and causes coagulation by simultaneous pressure that occurs as the probe is closed by the silicone boot at the other distal end [6, 7]. Nichrome wire at the ends of the probe cannot be activated via left foot switch unless completely squeezed by hands. Tissue-cutting is performed by clamping the coagulated tissue between the ends of the probe and activating via the right side of the foot switch (Figure 1).

In the present study, we performed extracapsular tonsillectomy with TWS using a probe with a power setting of “3” after placing the mouth gag. While the tonsil was retracted medially using allis clamp, dissection was made via tonsil probe at the upper pole of the tonsil by clamping the anterior pillar mucosal tissue. The tissue was activated with the foot switch and clamped between the ends of the tonsil probe for approximately 6 seconds and coagulation was performed. Thereafter, cutting procedure was performed by activating with the right foot switch for approximately two seconds. Dissection was extended from the upper pole to the lower pole by exposing the tonsil capsule. Tonsil tissue was coagulated for the last time at the lower pole and removed from the surgical area. This technique was performed for both tonsils. Tonsil bed was postoperatively monitored for bleeding. Surgical area was washed with normal saline. Cold diet was initiated both to the pediatric group and adult group 3 hours after the surgery. All of the patients were discharged on the postoperative 2nd day.

Pain was questioned by nurse on the 1st, 3rd, 7th, and 10th postoperative days via faces pain scale under the assistance of the families of the children younger than 8 years [8], whereas it was evaluated via visual analogue scale (VAS) in the patients older than 8 years [1]. Postoperative bleeding was monitored and recorded if any.

### 2.2. Statistical Analysis

Data were evaluated using the MedCalc statistics program version 11.5.1 (MedCalc Software, Mariakerke, Belgium). The Chi-square test was used to compare the categorical variables, whereas Mann-Whitney *U*-test and independent samples *t*-test were used to determine inter-group differences. The data were expressed as mean ± standard deviation. A *P* value <0.05 was considered significant. Comparison of two groups proportions, minimally required sample size per group 13.

## 3. Results

The present study included 213 patients, of whom 178 were pediatric and 35 were adult. The pediatric age group comprised 80 girls and 98 boys with a mean age of 6.7 ± 2.4 years (range 3–13 years). The adult age group comprised 20 male and 15 female patients with a mean age of 21.8 ± 7.07 years (range 15–41 years). Of the pediatric patients, 11 developed postoperative bleeding in the late term (1–10 days); bleeding was controlled under general anesthesia in two patients. Postoperative bleeding was developed in 7 of 35 adult patients in the late term, and the bleeding was controlled under general anesthesia in 3 patients. Postoperative bleeding was significantly less prevalent in the pediatric age group compared to the adult age group (*P* = 0.04). Moreover, postoperative pain was significantly less prevalent in the pediatric age group as compared to the adult age group (*P* < 0.001). Ages and postoperative pain in the pediatric and adult age groups are presented in Table 1.

## 4. Discussion

Tonsillectomy techniques have been compared through various parameters in the previous studies. These comparisons have been performed only among pediatric and/or adult age groups [3–5]. There have also been studies comparing various techniques in both age groups [2, 9]. However, limited number of studies in the literature has compared adult and pediatric patients in terms of the same technique. In the present study, we compared the adult and pediatric patients in terms of postoperative bleeding and pain following tonsillectomy performed TWS.

Karatzias et al. [9] reported no postoperative bleeding after tonsillectomy performed by TWS in the adult and pediatric patients. Moreover, operation duration was shorter in the pediatric patients than in the adults. In that particular study, 3 adult patients developed intraoperative bleeding from the tonsillar artery in the inferior pole region and the bleeding was controlled by bipolar electrocautery. In the present study, postoperative bleeding was developed in both groups, being significantly more prevalent in the adult age group. Karatzias et al. [9] mentioned that intraoperative bleeding requires additional coagulation of the large vessels and additional suture techniques particularly in the adult patients. This result was consistent with the result of the present study, which revealed more prevalent postoperative bleeding among adult patients.

Stavroulaki et al. [1] compared cold dissection and TWS in the adult patients and showed significantly lower pain scores in the TWS group particularly within the first four postoperative days. In the present study, pain scores were found higher in the adult age group as compared to the pediatric age group. Stavroulaki et al. [1] attributed this result to the fact that TWS was simple and faster and produced much lower collateral thermal damage as compared to the monopolar or bipolar electrocautery. They found no significant difference between these two techniques in terms of postoperative bleeding. Postoperative bleeding was observed only in 3 patients in the cold dissection group, of whom 2 had a history of peritonsillar abscess.

Karatzanis et al. [10] performed a comparative study in the adults using TWS and LigaSure method and found no difference between the techniques in terms of the intraoperative bleeding and mean operation duration. On the other hand, the mean pain score was significantly lower in the TWS group on each postoperative day as compared to that in the LigaSure group. Moreover, they noted late postoperative bleeding in 1 patient in the TWS group and in 2 patients in the LigaSure group. Thus, they concluded that both techniques provide adequate homeostasis in the adult patients. The present study showed higher incidence of postoperative bleeding among adult patients as compared to that in the TWS group of Karatzanis et al. [10].

In their comparative study conducted on adult patients using TWS and bipolar electrocautery, Karatzias et al. [3] reported that 9 patients presented with late postoperative bleeding, of whom 4 were in the TWS group and 5 were in the bipolar electrocautery group. They noted no bleeding in the mouths or pharynxes of 2 patients in the TWS group and 3 patients in the bipolar electrocautery group, and the patients were discharged. The remaining 1 (1.2%) patient in the TWS group and 3 (4.3%) patients in the bipolar electrocautery group were hospitalized because of bleeding in the oral cavity. Bleeding was controlled under general anesthesia in 1 patient in the bipolar electrocautery group. In the present study, postoperative bleeding was observed in 7 adult patients, of whom 4 (11.4%) were hospitalized due to bleeding in the oral cavity and were discharged without any intervention. Bleeding was controlled under general anesthesia in the remaining 3 (8.5%) patients.

In their multiparametric study, Chimona et al. [5] compared cold knife, radiofrequency, and TWS in the children undergoing tonsillectomy, and they reported that postoperative pain was significantly lower in the cold knife procedure. They found no significant difference between these three methods in terms of late postoperative bleeding. In that particular study, the incidence of postoperative bleeding was 2.23%, whereas, it was 6.18% among pediatric age group in the present study. Of the pediatric patients with postoperative bleeding, 2 (1.12%) required bleeding control under general anesthesia. Chimona et al. [5] indicated that the incidence of posttonsillectomy bleeding was low in their study and they attributed this result to the experience of the surgeons whom performed the procedure, as well as to the time that they recommended to the children to start their normal diet and activity.

Michel et al. [2] conducted a study on 100 patients and divided them into two subgroups according to their ages; patients between 2 and 12 years of age constituted younger group and patients between 13 and 47 years of age constituted older group. This study, which is the only study in the literature similar to the present study, introduced the preliminary results about safety, efficacy, and morbidity of TWS used for tonsillectomy and compared the results with those of other total tonsillectomy techniques in the literature. In that particular study, the mean pain score during healing was 2.0 in the younger group and 3.1 in the older group. These results were consistent with the results of the present study; the postoperative pain score was significantly lower in the pediatric patients compared to that in the adults. Moreover, in that particular study, the incidence of late postoperative bleeding was the same both in the younger and older groups (2%). The incidence of late postoperative bleeding in the present study was 6.18% in the pediatric age group and 20% in the adult age group.

In the literature, Lee et al. [11] reported that there was no difference between cold and hot dissections in terms of the incidence of secondary bleeding in the pediatric age group. Nevertheless, in their study Michel et al. [2] performed a literature review and reported the incidences of late postoperative bleeding for different techniques as follows: 8.6% (*n* = 1455) for electrodissection, 3.9% (*n* = 1829) for coblation, 2.8% (*n* = 468) for harmonic scalpel, and 1.5% (*n* = 1610) for cold knife. In the present study, the incidence of late postoperative bleeding was 6.18% in the pediatric age group and 20% in the adult age group.

The present study evaluated postoperative bleeding and pain following tonsillectomy performed by TWS in the pediatric and adult age groups and revealed higher incidences of postoperative bleeding and pain in the late term in the adult age group compared to those in the pediatric age group. Large-scale studies comparing different tonsillectomy techniques in adult and pediatric age groups are needed.

## Figures and Tables

**Table 1:** The ages and visual analogue scale scores of the patients.

	Mean ± SD	95% CI	RSD	Median	Min–Max
Pediatric patients (*n* = 178)					
Age	6.7 ± 2.40	6.3–7.0	0.35	6	2–13
VAS score	7.3 ± 1.15	7.2–7.5	0.15	8	6–10

Adult patients (*n* = 35)					
Age	21.8 ± 7.07	19.4–24.3	0.32	21	15–41
VAS score	85.6 ± 5.80	83.6–87.6	0.067	85	70–95

VAS: visual analogue scale; SD: standard deviation; CI: confidence interval; RSD: relative standard deviation; Min–Max: minimum–maximum.
